# c-Abl Activation Linked to Autophagy-Lysosomal Dysfunction Contributes to Neurological Impairment in Niemann-Pick Type A Disease

**DOI:** 10.3389/fcell.2022.844297

**Published:** 2022-03-18

**Authors:** Tamara Marín, Andrés E. Dulcey, Fabián Campos, Catalina de la Fuente, Mariana Acuña, Juan Castro, Claudio Pinto, María José Yañez, Cristian Cortez, David W. McGrath, Pablo J. Sáez, Kirill Gorshkov, Wei Zheng, Noel Southall, Maria Carmo-Fonseca, Juan Marugán, Alejandra R. Alvarez, Silvana Zanlungo

**Affiliations:** ^1^ Department of Gastroenterology, Faculty of Medicine, Pontificia Universidad Católica de Chile, Santiago, Chile; ^2^ Early Translation Branch, National Center for Advancing Translational Sciences (NCATS), NIH, Rockville, MD, United States; ^3^ Laboratory of Cell Signaling, Center for Aging and Regeneration (CARE), Millennium Institute on Immunology and Immunotherapy (IMII), Department of Cellular and Molecular Biology, Biological Sciences Faculty, Pontificia Universidad Católica de Chile, Santiago, Chile; ^4^ Department of Medicine, Brigham and Women’s Hospital, Harvard Medical School, Boston, MA, United States; ^5^ School of Medical Technology, Health Sciences Faculty, Universidad San Sebastián, Santiago, Chile; ^6^ Center for Genomics and Bioinformatics, Faculty of Science, Universidad Mayor, Santiago, Chile; ^7^ Cell Communication and Migration Laboratory, Institute of Biochemistry and Molecular Cell Biology, Center for Experimental Medicine, University Medical Center Hamburg-Eppendorf, Hamburg, Germany; ^8^ Instituto de Medicina Molecular Joȧo Lobo Antunes, Faculdade de Medicina, Universidade de Lisboa, Lisbon, Portugal

**Keywords:** Niemann-Pick disease, neurodegeneration, c-Abl kinase, autophay-lysosomal pathway, lysosomal storage disorder (LSD)

## Abstract

Niemann-Pick type A (NPA) disease is a fatal lysosomal neurodegenerative disorder caused by the deficiency in acid sphingomyelinase (ASM) activity. NPA patients present severe and progressive neurodegeneration starting at an early age. Currently, there is no effective treatment for this disease and NPA patients die between 2 and 3 years of age. NPA is characterized by an accumulation of sphingomyelin in lysosomes and dysfunction in the autophagy-lysosomal pathway. Recent studies show that c-Abl tyrosine kinase activity downregulates autophagy and the lysosomal pathway. Interestingly, this kinase is also activated in other lysosomal neurodegenerative disorders. Here, we describe that c-Abl activation contributes to the mechanisms of neuronal damage and death in NPA disease. Our data demonstrate that: 1) c-Abl is activated *in-vitro* as well as *in-vivo* NPA models; 2) imatinib, a clinical c-Abl inhibitor, reduces autophagy-lysosomal pathway alterations, restores autophagy flux, and lowers sphingomyelin accumulation in NPA patient fibroblasts and NPA neuronal models and 3) chronic treatment with nilotinib and neurotinib, two c-Abl inhibitors with differences in blood-brain barrier penetrance and target binding mode, show further benefits. While nilotinib treatment reduces neuronal death in the cerebellum and improves locomotor functions, neurotinib decreases glial activation, neuronal disorganization, and loss in hippocampus and cortex, as well as the cognitive decline of NPA mice. Our results support the participation of c-Abl signaling in NPA neurodegeneration and autophagy-lysosomal alterations, supporting the potential use of c-Abl inhibitors for the clinical treatment of NPA patients.

## Introduction

Mutations in the *SMPD1* gene that encodes for acid sphingomyelinase (ASM), a key lysosomal enzyme that hydrolyzes sphingomyelin to ceramide and phosphocholine, lead to Niemann Pick type A (NPA) disease (Schuchman, 2007). NPA disease is a fatal lysosomal neurodegenerative disorder characterized by sphingomyelin accumulation in lysosomes leading to lysosomal dysfunction and autophagy alterations (Yanez et al., 2020). NPA patients present with developmental delay, hepatosplenomegaly, and progressive neurodegeneration that initially affects Purkinje neurons in the cerebellum (Otterbach and Stoffel, 1995). Additionally, the brain shows astrogliosis, and the astrocytes in the NPA hippocampus and cortex present altered morphology (Perez-Canamas et al., 2017). Unfortunately, NPA patients typically die between 2 and 3 years of age (Schuchman and Desnick, 2017).

The mechanisms that lead to neuronal death in NPA disease are not fully understood. Previous work recognized the role of calcium imbalance (Perez-Canamas et al., 2017), neuronal endocannabinoid system alterations (Bartoll et al., 2020), and autophagy alterations (Gabande-Rodriguez et al., 2014; Li et al., 2014). Many neurodegenerative disorders are associated with autophagy alterations, reflecting the contribution of this process to neuronal physiology as it is involved in quality control of cytosolic components, such as damaged proteins and organelles, survival, and differentiation (Nixon, 2013; Lee et al., 2016; Menzies et al., 2017). Recent studies have shown autophagy-lysosomal pathway (ALP) alterations in NPA disease at different levels, including autophagosome-lysosome fusion (Li et al., 2014) and lysosomal membrane permeabilization (Gabande-Rodriguez et al., 2014). Therefore, pharmacological and genetic manipulations to promote better functioning of the ALP in clearing accumulated materials may be therapeutic in neurodegenerative disorders, including NPA disease.

c-Abl is a non-receptor tyrosine kinase that has different biological functions depending on the cell type and regulates several pathways including apoptosis and ALP, in response to different signals. c-Abl has been reported as a central signaling kinase in different neurodegenerative disorders including Alzheimer’s (Alvarez et al., 2004; Cancino et al., 2008), Parkinson’s (Ko et al., 2010), Amyotrophic lateral sclerosis (ALS) (Imamura et al., 2017) and Niemann Pick type C (NPC) disease (Klein et al., 2011; Contreras et al., 2020), among others (Yanez et al., 2020). Recently, c-Abl has been shown to be involved in autophagy. Chronic treatment with nilotinib, a clinically validated c-Abl inhibitor, improves autophagy, reduces Aβ levels, and prevents neurodegeneration in an Alzheimer’s mouse model (La Barbera et al., 2021). In Parkinson’s, nilotinib induces cellular clearance of α-synuclein, via autophagic degradation, and protects the dopaminergic neurons, improving locomotor function in mouse models of this disease (Hebron et al., 2013). In addition, c-Abl inhibition using a classic inhibitor, imatinib, induces autophagy through the overexpression of genes involved in this process (Can et al., 2011). Interestingly, c-Abl kinase regulates ALP through transcription factor EB (TFEB) modulation (Contreras et al., 2020), the master transcriptional factor that drives the expression of genes related to autophagy and lysosomal biogenesis, and exocytosis.

The relation between c-Abl kinase activation, neurodegeneration, and the ALP in NPA disease has not been explored yet. In this work, we show that c-Abl signaling participates in the pathogenic mechanisms leading to neurodegeneration in NPA disease. We found that c-Abl is activated in several NPA models and its inhibition promotes the improvement in the ALP inducing autophagy flux and reducing sphingomyelin accumulation *in vitro* NPA models. Moreover, c-Abl inhibition decreases neuronal death, astrogliosis, inflammation markers, neuronal disorganization, and improves locomotor and cognitive functions in NPA mice.

## Material and Methods

### NPA Models

NPA cellular models: i) Primary skin fibroblasts from an NPA patient (GM13205) carrying one mutation at the *SMPD1* gene were purchased from the Coriell Institute for Medical Research. These NPA fibroblasts have no detectable sphingomyelinase activity; donor subject (female Ashkenazi, 2 years old) had one allele with a deletion of a single cytosine in exon 2 at codon 330 of the *SMPD1* gene [990delC] resulting in a frameshift leading to the formation of a premature stop (TGA) at codon 382 [P330fsX382]. Fibroblasts from an unaffected individual (HC; healthy control) (GM05659) were used as control; ii) Primary cultures of neurons were prepared from the hippocampus of wild-type (WT) and NPA mouse embryos age E18 and kept in culture for 7 days as described by Kaech et al. (2006). iii) NPA Neural Stem cells (NPA NSC) derived from NPA fibroblasts (GM16195) which were previously described (Long et al., 2016) and iv) SH-SY5Y cells were treated with desipramine 5, 10, 20 μM by 24 h to mimic the NPA phenotype.

NPA mice (ASMKO; *Smpd1* ^−/−^): They were created by gene targeting, as described previously (Horinouchi et al., 1995) and were kindly donated by Dr. Fernández-Checa (University of Southern California Research Center for Alcoholic Liver and Pancreatic Diseases and Cirrhosis, Keck School of Medicine, University of Southern California, Los Angeles, CA, United States and Cell Death and Proliferation, Institute of Biomedical Research of Barcelona (IIBB), CSIC, Barcelona, España). *Smpd1* ^-/-^ mice were obtained using heterozygous C57BL/6 breeding pairs and the littermates were used as controls. Animals were maintained in the Animal Care Facility of Pontificia Universidad Católica de Chile. Genotypes were identified using a PCR-based screening as described previously (Horinouchi et al., 1995). All procedures were approved by the ad hoc committee of Chile (ANID) and the Institutional Animal Care and Bioethical and Biosafety Committee of the UC (Protocol #170912002).

### Cellular and Animal Treatments

Human fibroblasts and NSC were maintained in Dulbecco’s modified Eagle’s medium (DMEM) supplied with 15% fetal bovine serum (FBS). Primary neurons were maintained in Neurobasal supplemented with 2% B27, 2 mM glutamine, 100 U/ml penicillin, and 100 μg/ml streptomycin. The proliferation of non-neuronal cells was limited using cytosine arabinoside 1 µM. Cells were treated with imatinib for 24 h, the concentration used was dependent on each cellular type; fibroblasts were treated with 10 μM, primary neurons with 5 μM, and NSCs with 100 nM of imatinib.

Acute treatment with a c-Abl inhibitor: Male/female WT and NPA mice received daily intraperitoneal injections (i.p.) of imatinib mesylate (Novartis, Basel, Switzerland) 12.5 mg/kg in 0.9% NaCl from postnatal day 21 (P21) to P49. Control groups (WT and NPA) received daily intraperitoneal injections of 0.9% NaCl. Bodyweight was measured twice a week during the full period of treatment, as well as locomotors tests were realized once a week.

Chronic treatment with c-Abl inhibitors: Male/female WT and NPA mice received diets supplemented with the c-Abl inhibitor nilotinib or neurotinib *ad libitum* from P21 until 5, 7, and 11 months of age. Control groups received a control diet.The rodent chow diet was manufactured by Envigo/Teklad by incorporation of neurotinib 67 ppm (10 mg/kg) or nilotinib at 200 ppm (30 mg/kg) into the NIH-31 Open Formula Mouse/Rat Sterilizable Diet (7017), followed by irradiation handling of the final product. Animal bodyweight was measured twice a week during the full period of treatment. The distribution of male/females in the control and treatment groups was 60/40%. No gender-dependent differences were observed in any of the results.

### Immunofluorescence Analysis of Cultured Cells

Fibroblasts and primary neurons were seeded on poly-lysine-coated coverslips (30,000 cells/cover). After treatment, cells were fixed in 4% paraformaldehyde/4% sucrose in PBS and permeabilized with 0.02% Triton X-100. Then, cells were blocked with 3% bovine serum albumin in PBS. Immunostaining was carried out using anti-tyrosine 412 phosphorylated of c-Abl (Y412) (anti-p-c-Abl) (C5240, Sigma Chemical co), anti-p62 (ab56416; Abcam), anti-LAMP1 (1D4B, sc-19992; Santa Cruz Biotechnology). Anti-rabbit IgG conjugated with Alexa Fluor-488 and anti-mouse IgG conjugated to Alexa Fluor-555 and Hoechst 33342 (H3570) were obtained from Invitrogen Detection Technologies. Fluorescent images were captured with an Olympus BX51 microscope (Olympus, Tokyo, Japan) and analyzed with the Image-Pro Express program (Media Cybernetics). We examined at least five images by cover and three covers by condition were stained by experiment in at least three independent experiments.

### BODIPY-SM and Filipin Staining in Cells

Briefly, 20,000 cells/well were seeded on coverslips on 24-well plates after 4 h incubation at 37°C with 5% CO_2_, 0.2 mg/ml BODIPY-FL C122 sphingomyelin (BODIPY-SM; catalog no. D7711, Thermo Fisher Scientific) was added to cells, and incubated overnight. Then, cells were fixed with 4% paraformaldehyde solution. Later, cells were incubated with 1 mg/ml Hoechst 33342 (H3570; Invitrogen) in PBS with incubation at room temperature for 10 min. After washing, covers were mounted with Fluoromount-G, and cells were imaged in the Olympus BX51 microscope (Olympus).

For Filipin staining, cells were fixed in 4% paraformaldehyde/4% sucrose in PBS for 30 min. After, cells were washed with PBS and treated with 1.5 mg/ml glycine for 20 min. Finally, cells were treated with 25 μg/mL Filipin (F-8765, Sigma Chemical Co.) for 30 min, washed with PBS and covered with Fluoromount-G. Images were captured with an Olympus BX51 microscope.

### mRFP-GFP Tandem Fluorescent-Tagged LC3 Expression

Neural Stem Cells were transduced with 30 particles per cell of the mRFP-GFP tandem fluorescent-tagged LC3 (Premo™ Autophagy Tandem Sensor RFP-GFP-LC3B, P36239, Thermo Fisher Scientific), as described in the manufacturer’s instructions. After 24 h, cells were rinsed in 1 × PBS, nuclei were stained with Hoechst 33342 (H3570; Invitrogen) and processed for analysis in an LSM510 META microscope (Carl Zeiss AG). Quantification of only RFP-positive dots or dots positive for GFP and RFP was performed with ImageJ software.

### Western Blot Analysis

Proteins were prepared as described previously (Cancino et al., 2008). Tissue protein samples (30 μg) and cellular protein samples (50 μg) were resolved by SDS–PAGE. The immunoblot was carried out using anti-c-Abl (A5844, Sigma-Aldrich, USA), p-c-Abl (Tyr412) (07–788; Millipore), anti-LC3 (NB100-2220), anti-p62 (ab56416), and anti-GAPDH (0411; sc47724; Santa Cruz Biotechnology) antibodies. The secondary antibodies against rabbit or mouse IgGs conjugated with horseradish peroxidase were obtained from Upstate Biotechnology, Lake Placid, NY, United States.

### Hanging and Memory Flexibility Test

During the treatments, locomotor coordination was evaluated through the Hanging test. The mouse was placed at the center of a horizontal bar (3 mm diameter; 35 mm long) hanging with its forepaws. The body position of the animal was observed for 30 s and scored as previously described (Voikar et al., 2002)

Spatial memory acquisition and learning of animals was assessed using the modified Morris water maze test called Memory flexibility test (Chen et al., 2000; Toledo and Inestrosa, 2010) which consisted of a dark blue plastic pool 100 cm in diameter and 40 cm in depth, located in a 2.5 × 2.5-m room with numerous extra-maze visual cues that remained constant throughout the experiment. The pool was filled with water (a depth of 28 cm) and a clear acrylic glass platform (10 cm in diameter and 26 cm high) was positioned in the pool and its location was changed every day during the test. Testing was performed for four consecutive days. Each day the animal completed 15 swim trials to find the platform, each trial for 40 s. The animal reaches the acceptable memory criteria when it reaches the platform in three consecutive trials in less than 20 s per attempt. A minimum of 5 min is expected between trials per animal. A mean of 15 trials to reach the platform for each mouse were used in the statistical analyses.

### Tissue Immunohistochemical and Immunofluorescence Procedures

Mice were anesthetized with xylazine 0.12 mg/10 g and ketamine 0.8 mg/10 g and intracardially perfused with 0.9% NaCl. Then, the cerebellum and brain were removed and postfixed with 4% paraformaldehyde in PBS overnight, followed by 30% sucrose in PBS at 4°C overnight. Cerebella were cut in 30 μm sagittal sections, and brains were cut 20 μm coronal sections with a cryostat (Leica CM 1850) at −20°C. 2–3 slices by animal were stained by experiment. We examined at least three animals per condition for quantitative analysis. For immunohistochemistry, slices were treated with H_2_O_2_ 0.3% for 30 min, washed four times with PBS, treated with NaBH_4_ 10 mg/ml for 15 min, washed with PBS three times by 10 min, and blocked with BSA 0.5% triton x-100 0.2% for 1 h. Anti-calbindin D-28K antibody (AB1778, Chemicon International), anti-NeuN (ab177487, Abcam) were used with the avidin-biotin-horseradish peroxidase complex method (Vector Laboratories, Burlingame, CA, United States). Entellan was used as mounting medium.

For immunofluorescence, slices were treated with 0.4% triton x-100 for 30 min, glycine 0.15 M for 15 min, NaBH_4_ 10 mg/ml for 15 min, washed with PBS three times by 10 min, and blocked with BSA 3% triton x-100 0.4% for 1 h. We used anti-GFAP (#3670, Cell signaling technology), anti-Iba-1 (NB100-1028, NovusBio), anti-CD68 (MCA1957GA; Bio-Rad), and Hoechst 33,342 (H3570, Invitrogen). Secondary antibodies anti-rabbit IgG conjugated with Alexa Fluor-488, anti-mouse IgG conjugated to Alexa Fluor-555, and anti-rat IgG conjugated with FITC were obtained from Invitrogen Detection Technologies. Fluoromount-G was used as mounting medium. Images were captured with an Olympus BX51 microscope (Olympus) and analyzed with the Image-Pro Express program (Media Cybernetics, Bethesda, MD, United States).

Analysis of astrocyte and microglia size and shape were performed in Fiji ImageJ from fluorescent images acquired in a DMi8 Leica microscope, with a PL FLUOTAR 40 × with a numerical aperture of 0.80. Then, images were analyzed by using a custom-made macro based on the principles previously described in other cellular systems (Saez et al., 2018). Briefly, after background subtraction, the maximal z-projection was obtained from the planes that contained the cells. Then, semi-automatic segmentation was performed by using Li’s threshold method and manual post-correction of the region of interest. Shape descriptors, such as area and solidity [defined as: (Area/Convex area)], were calculated from segmented images.

### Filipin Staining in Tissue

Slices were treated with NaBH_4_ 10 mg/ml for 10 min. Then, slices were incubated with Filipin (F-8765, Sigma Chemical Co.) overnight. The next day, slices were washed with 1 × PBS and mounted with gelatin 0.1%. Fluoromount-G was used as mounting medium.

### Statistical Analysis

Mean and standard error with the corresponding number of experiments are indicated in each figure legend. Probability values of the data for Student *t*-tests and ANOVA followed by Tukey *post-hoc* test were calculated using GraphPad Prism 8 (Graph Pad Software, Inc., San Diego, USA).

In the box-and-whisker plots, the center line denotes the median value, edges are upper and lower quartiles, whiskers show minimum and maximum values and points are individual experiments or number of animals or cells.

## Results

### c-Abl Is Active and its Inhibition Decreases Autophagy and Lysosomal Alterations in NPA Patient Fibroblasts

**FIGURE 1:**
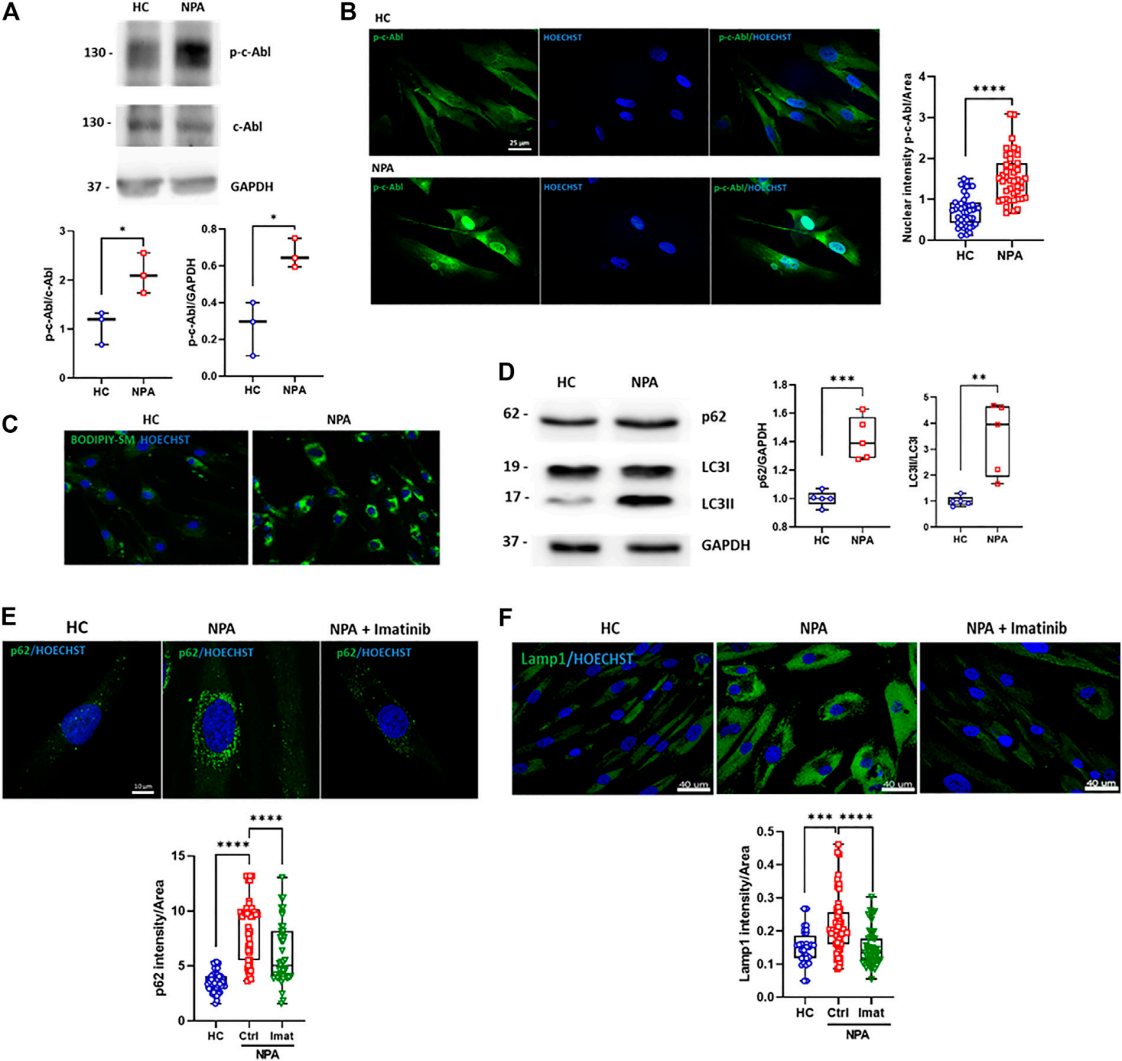
c-Abl activation regulates autophagy and lysosomal alterations in NPA fibroblasts. **(A)** Human fibroblasts homogenates from NPA patient and healthy control (HC) subjects (50 μg protein/lane) were used to measure p-c-Abl levels. The graph shows quantifications of p-c-Abl levels normalized by GAPDH and c-Abl expression. The data shown are from three independent experiments. Student’s *t*-test: *p < 0.05. **(B)** HC and NPA fibroblasts were fixed and immunostained using an anti-p-c-Abl Tyr412 antibody (green) and Hoechst staining for nucleus (blue). For each condition, *n* = 15 cells were measured by experiment; three independent experiments were performed. Student’s *t*-test: ****p < 0.0001. **(C)** HC and NPA fibroblasts were incubated with BODIPY-SM to confirm sphingomyelin accumulation. The images were taken with a × 40 objective lens. **(D)** Homogenates from fibroblasts from NPA patient and healthy control (HC) subject (50 μg protein/lane) were used to measure p62 and LC3II levels. The graph shows quantifications of protein levels normalized by GAPDH and LC3I expression, respectively. The image is representative of five independent experiments. Student’s *t*-test: **p < 0.01; ***p < 0.001; **(E)** HC and NPA fibroblasts were treated with imatinib (10 μM) for 24 h, fixed, and immunostained using an anti-p62 antibody (green) and **(F)** anti-Lamp1 (green). Hoechst staining for the nucleus (blue). For each condition, *n* = 10–18 cells were measured by experiment; three independent experiments were performed. ANOVA, Tukey *post-hoc*: ***p < 0.001; ****p < 0.0001. In the box-and-whisker plots, the center line denotes the median value, edges are upper and lower quartiles and whiskers show minimum and maximum values.

c-Abl contributes to other neurodegenerative disorders linked to ALP alterations (Ren et al., 2018; Contreras et al., 2020). When this kinase is activated, it is phosphorylated at tyrosine 412 (p-c-Abl) and also partially changes its location from the cytosol to the nucleus. To evaluate if c-Abl is active in NPA disease, we analyzed the levels of p-c-Abl by Western blot in GM13205 NPA patient fibroblasts (NPA fibroblasts), which harbor one of the most common mutations in NPA disease (see material and methods section). Interestingly, we found that the p-c-Abl levels are increased in comparison to fibroblasts from a healthy subject (HC fibroblasts; GM05659) (Figure 1A). Moreover, we observed that p-c-Abl kinase is translocated to the nucleus in NPA fibroblasts (Figure 1B; Supplementary Figure S1A). These results show that c-Abl kinase is activated in fibroblasts from an NPA patient.

In order to test the involvement of c-Abl in NPA disease ALP alterations, we characterized NPA fibroblasts. In agreement with previous reports, NPA fibroblasts showed sphingomyelin accumulation (Figure 1C). We found that the autophagy and lysosomal markers, p62 and LC3II levels, were increased in NPA fibroblasts (Figure 1D), confirming autophagy alterations. Furthermore, immunofluorescence analysis showed an accumulation of p62 positive-autophagic vesicles around the nucleus (Figure 1E; Supplementary Figure S1B). Also, we found that Lamp1 levels were increased, confirming that NPA fibroblasts contain more acid vesicles and lysosomes than HC fibroblasts (Figure 1F; Supplementary Figure S1C).

Interestingly, when we treated NPA fibroblasts with imatinib, a classic c-Abl inhibitor, we found a significant decrease in the number of p62-positive vesicles. Moreover, imatinib treatment restored the p62-positive vesicles distribution (Figure 1E; Supplementary Figure S1B). A similar trend was observed for the Lamp1 signal, which decreased when NPA fibroblasts were treated with imatinib (Figure 1F; Supplementary Figure S1C). These results show that NPA fibroblasts present autophagy and lysosomal alterations and suggest that c-Abl activation is regulating them.

### c-Abl Inhibition Improves Autophagy Flux and Decreases Sphingomyelin Accumulation in NPA Neuronal Models

**FIGURE 2:**
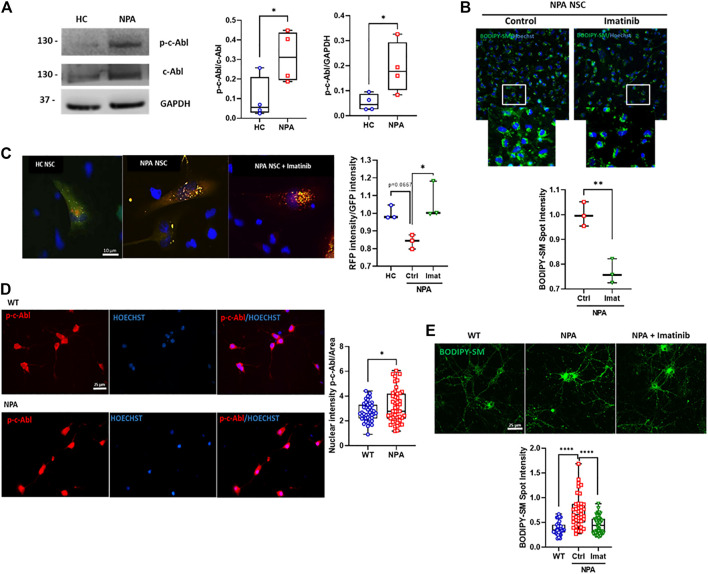
c-Abl inhibition improves autophagy flux and decreases sphingomyelin accumulation in NPA neuronal models. **(A)** p-c-Abl levels were measured in Neural Stem Cells (NSCs) extracts by Western blot. Images representative from four independent experiments are shown. Student’s *t*-test: *p < 0.05. **(B)** BODIPY-SM staining indicates sphingomyelin accumulation. Fluorescent microscopic images of NPA NSCs treated imatinib 0.001 µM by 24 h. For each condition, *n* = 150 cells were measured by experiment; three independent experiments were performed. Student’s *t*-test: **p < 0.01. **(C)** NPA NSCs expressing mRFP-GFP-LC3 were treated with imatinib 0.001 µM by 24 h or vehicle. Graph shows the rate between RFP intensity and GFP intensity corresponding to autolysosomes. For each condition, *n* = 50 cells were measured by experiment; three different experiments were performed. Student’s *t*-test: *p < 0.05. **(D)** Primary neurons were 7 days *in vitro*, fixed, and immunostained using anti-p-c-Abl Tyr412 antibody (red) and Hoechst staining for nucleus (blue). For each condition, *n* = 10–20 neurons were measured by experiment; three independent experiments were performed. Student’s *t*-test: *p < 0.05. **(E)** Primary hippocampal neurons were treated with imatinib 5 µM by 24 h. Sphingomyelin accumulation was analyzed by BODIPY-SM. For each condition, *n* = 10–20 neurons were measured by experiment; three independent experiments were performed. ANOVA, Tukey *post-hoc*: ****p < 0.0001. In the box-and-whisker plots, the center line denotes the median value, edges are upper and lower quartiles and whiskers show minimum and maximum values.

Next, we explored the activation of c-Abl in a neuronal NPA model using NPA Neural Stem cells (NPA NSC) derived from NPA fibroblasts (GM13205). The NPA NSCs exhibit a disease phenotype of lysosomal sphingomyelin accumulation and enlarged lysosomes and can be used as a cell-based disease model for studying the disease pathophysiology (Long et al., 2016). Interestingly, in this human neuronal NPA model, we also found that the levels of activated c-Abl are increased compared to NSCs derived from healthy individual fibroblasts (HC NSCs) (Figure 2A). We also confirmed c-Abl activation in another neuronal pharmacological NPA model, treating SH-SY5Y neuronal cells with the ASM inhibitor desipramine (Kornhuber et al., 2010). We confirmed the NPA phenotype through lipid accumulation using Filipin staining indicating the secondary accumulation of cholesterol (Supplementary Figure S2A) and a significant reduction in ASM activity in desipramine treated cells compared to control cells (Supplementary Figure S2B). Interestingly, we found an increase of p-c-Abl levels in SH-SY5Y cells treated with desipramine (Supplementary Figure S3A) and p-c-Abl nuclear localization in desipramine-treated cells (Supplementary Figure S3B).

To assess the contribution of c-Abl activity to the autophagy flux and cellular clearance dysfunction, we treated NPA NSCs with imatinib for 24 h and we evaluated sphingomyelin accumulation using BODIPY-SM. We found that imatinib treatment induces a decrease in sphingomyelin accumulation in NPA NSCs (Figure 2B).

We further analyzed the effect of c-Abl inhibition on autophagy flux using the Premo™ Autophagy Tandem Sensor RFP-GFP-LC3B in NPA NSCs. Cells incubated with this sensor express an LC3 fusion protein fused to an acid-sensitive GFP and an acid-insensitive RFP. The expression of this LC3 fusion protein allows visualizing the progression from autophagosome (neutral pH) to autolysosome (with an acidic pH) through the specific loss of the GFP fluorescence. Interestingly, HC NSCs mainly showed a diffuse green signal while NPA NSCs showed both punctuate GFP and RFP fluorescence (yellow colocalization), suggesting autophagosomes accumulation due to a decrease in autophagosome-lysosome fusion in NPA (Figure 2C). Furthermore, the ratio between red intensity/green intensity was significantly increased with imatinib treatment in comparison to untreated NPA NSCs, suggesting that imatinib increases autolysosome formation (Figure 2C) thereby inducing autophagosome-lysosome fusion and increasing autophagy flux.

In addition, we analyzed c-Abl activation in primary cultures of hippocampal neurons obtained from the NPA mouse model, which was developed by the targeted deletion of the gene that codifies ASM (Horinouchi et al., 1995). The NPA mouse model (ASMKO; *Smpd1* ^*−/−*^) does not have residual ASM activity and exhibits progressive lipid storage in the reticuloendothelial (RES) organs, as well as in the brain (McGovern et al., 2017). First, we analyzed p-c-Abl levels by immunofluorescence from primary neurons from WT and NPA mouse embryos. We found that p-c-Abl levels and nuclear localization were increased in NPA primary neurons compared to WT neurons (Figure 2D; Supplementary Figure S1D). Moreover, we also found that imatinib treatment decreased sphingomyelin accumulation in NPA primary neurons (Figure 2E; Supplementary Figure S1E). These results show that c-Abl inhibition lowers sphingomyelin accumulation and suggests that c-Abl inhibition improves cellular clearance and autophagy flux in neuronal NPA models.

### Acute c-Abl Inhibition Decreases the Neuronal Death in Cerebellum and Improves Locomotor Function in NPA Mice

**FIGURE 3:**
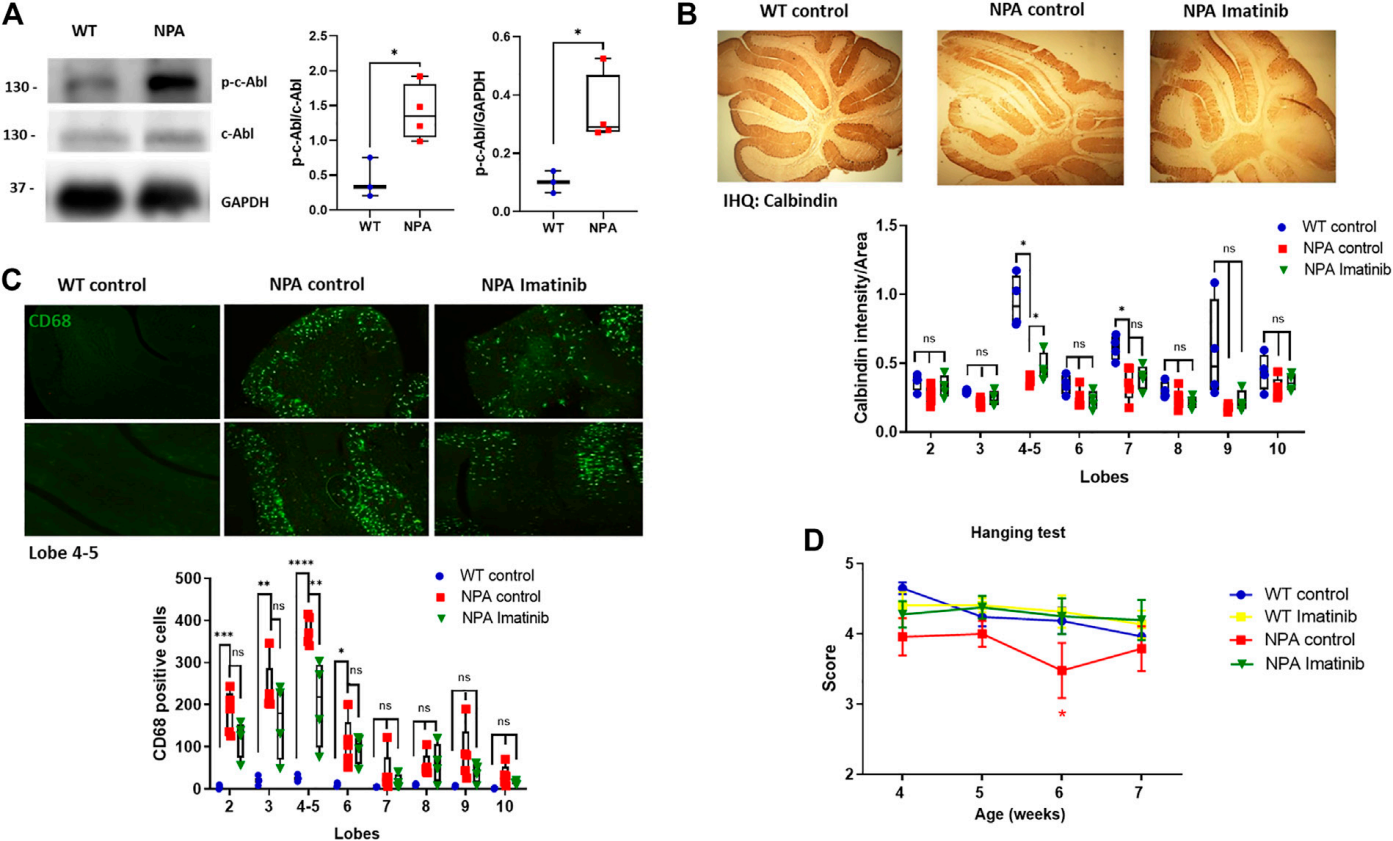
Acute imatinib treatment decreases neuronal death in the cerebellum and improves locomotor function in NPA mice. **(A)** WT and NPA brain homogenates (50 μg protein/lane) from mice at 4 weeks old were analyzed by Western blot. The graph shows quantifications of p-c-Abl levels normalized by GAPDH and c-Abl levels. The number of animals was: WT = 3; NPA = 4; Student’s *t*-test: *p < 0.05. **(B)** WT and NPA mice were i.p. injected with imatinib (12.5 mg/kg) or vehicle from 3 weeks of age until 7 weeks of age. The Purkinje neuron marker calbindin was analyzed by immunohistochemistry. A quantification of calbindin-immunoreactive Purkinje cell bodies in cerebellar sections is shown. **(C)** CD68 marker was evaluated by immunofluorescence analysis in the cerebellum from WT and NPA mice. For **(B,C)**, the number of animals was WT control = 3; NPA control = 5; NPA imatinib = 4. Images were taken with × 4 objective. ANOVA, Tukey *post-hoc:* *p < 0.05; **p < 0.01; ***p < 0.001; ****p < 0.0001 **(D)** Mice were treated for 4 weeks and motor coordination was assessed weekly by the hanging test. Data are shown as mean ± SEM. ANOVA, Tukey *post-hoc*: *p < 0.05, NPA control is statistically different from WT control and NPA with imatinib. The following number of animals was used: WT control = 11; WT imatinib = 10; NPA control = 8; NPA imatinib = 9. In the box-and-whisker plots, the center line denotes the median value, edges are upper and lower quartiles, whiskers show minimum and maximum values and points are individual experiments.

To evaluate the relevance of c-Abl activity in neurodegeneration, we used the NPA mouse model described above, which exhibits progressive degeneration of Purkinje neurons in the cerebellum, gliosis, and demyelination (Otterbach and Stoffel, 1995). After we confirmed that c-Abl is active through Western blot in the central nervous system (CNS) in NPA mice (Figure 3A), we used an acute and short treatment scheme to evaluate the neuronal progression of NPA pathology. WT and NPA mice were injected intraperitoneally (i.p.) daily with imatinib (12.5 mg/kg) or vehicle from three until 7 weeks of age. The Purkinje cells loss was followed by immunohistochemistry analysis against calbindin. We found that the cerebellum of NPA mice showed less calbindin staining than WT mice at anterior lobules, specifically 4–5, indicating Purkinje neurons loss (Figure 3B). This result is supported by previous studies that show that the loss of Purkinje neurons starts from the anterior lobes of the cerebellum and at older ages loss occurs in the posterior lobes (Macauley et al., 2008). Interestingly, the NPA mice treated with imatinib showed an increased calbindin staining, suggesting an improvement in the survival of the Purkinje neurons. A significant effect was found at lobules 4–5 (Figure 3B). Also, we evaluated the levels of the microglia marker CD68 by immunofluorescence. The cerebellum of NPA mice showed higher CD68 levels than the cerebellum of WT mice at anterior lobes and imatinib treatment significantly decreased CD68 positive cells specifically in lobules 4–5 compared to NPA mice cerebellum (Figure 3C).

Furthermore, we evaluated coordination and locomotor skills using the Hanging test (Voikar et al., 2002). We found that NPA mice showed impairment of the locomotor function compared to WT mice, whereas NPA mice treated with imatinib improved locomotor function in comparison to control NPA mice (Figure 3D). We did not find any difference in mice’s gain of weight with the imatinib treatment (Supplementary Figure S4).

### Chronic c-Abl Inhibition Treatment Delays Locomotor Impairment in NPA Mice

**FIGURE 4:**
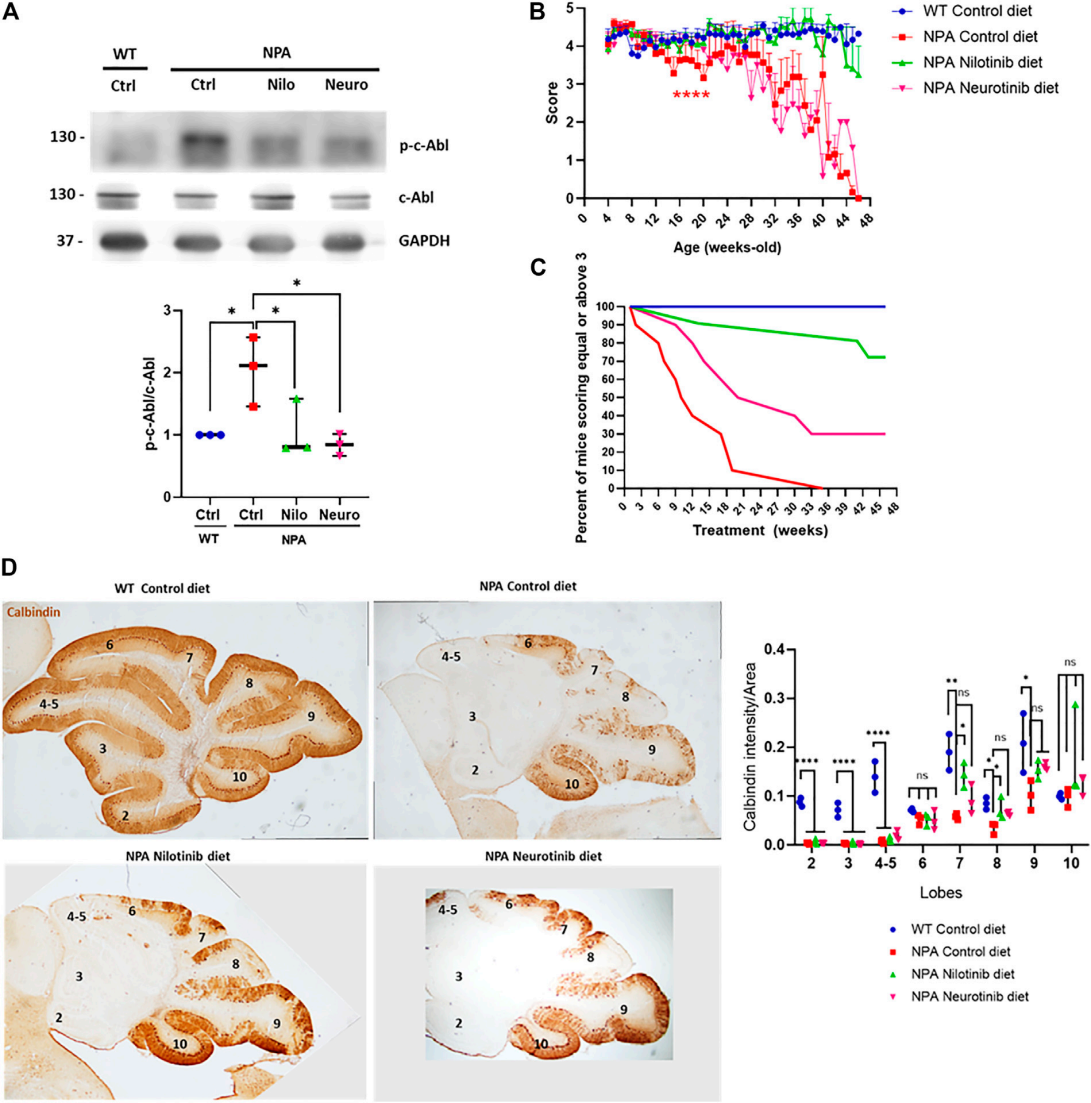
Chronic c-Abl inhibition treatment delays locomotor impairment in NPA mice. WT and NPA mice received nilotinib (200 ppm; 30 mg/kg) and neurotinib (67 ppm; 10 mg/kg) supplemented diets or control diet starting at p21 until 11 months of age. **(A)** p-c-Abl protein levels were evaluated in cerebellum homogenates from WT and NPA mice of 5 months of age by Western blot. The number of animals was three by condition. ANOVA, Tukey *post-hoc*:*p < 0.05. **(B)** Motor coordination was assessed weekly by the Hanging test. Data are shown as mean ± SEM. ANOVA, Tukey *post-hoc*: ****p < 0.0001; NPA control is statistically different from WT control and nilotinib NPA. **(C)** Deterioration curve of mice was performed using percent of mice with a score equal to or above 3. For **(B,C)**, the following number of animals was used: WT control (Ctrl) = 10; NPA control (Ctrl) = 10; NPA nilotinib (Nilo) = 11; NPA neurotinib (Neuro) = 10. **(D)** Purkinje neuron marker Calbindin was analyzed by immunohistochemistry. Calbindin intensity was quantified. A representative image by condition is shown (*n* = 3 mice/group). Images were taken with × 2 objective. ANOVA, Tukey *post-hoc:* *p < 0.05; **p < 0.01; ***p < 0.001; ****p < 0.0001. In the box-and-whisker plots, the center line denotes the median value, edges are upper and lower quartiles, whiskers show minimum and maximum values and points are the number of animals used.

Our data above show that c-Abl inhibition decreases neuronal death in NPA mice at early ages. However, considering that NPA mice live for approximately 11 months, we decided to employ a longer, chronic, and less invasive treatment approach. We treated the animals with nilotinib and neurotinib-supplemented diets starting at 3 weeks of age. Both neurotinib and nilotinib are c-Abl inhibitors but these inhibitors have different mechanisms and brain penetration. Nilotinib binds to the ATP binding cleft between the N-terminal and C-terminal lobes, while neurotinib binds to an allosteric pocket for myristate at the C-terminal lobe of the kinase domain (Greuber et al., 2013; Wang, 2014). Nilotinib has been used in clinical trials for different neurodegenerative pathologies such as Parkinson’s disease (Abushouk et al., 2018; Pagan et al., 2019) and Alzheimer’s disease (Turner et al., 2020). Neurotinib is a new drug developed by our group in collaboration with the National Center for Advancing Translational Sciences at the National Institutes of Health (NCATS-NIH) which has favorable potency, selectivity, pharmacokinetics, and vastly improved central nervous system permeability that reaches higher concentration in the brain than nilotinib (Supplementary Figure S5B). The pharmacokinetic characterization of neurotinib is shown in Supplementary Figure S5. Animals were fed with a control diet or diets supplemented with neurotinib (67 ppm; 10 mg/kg) and nilotinib (200 ppm; 30 mg/kg) from 3 weeks of age to 11 months of age. We observed that p-c-Abl protein levels are increased in the cerebellum of control diet-treated NPA mice in comparison to WT mice with the same diet, indicating c-Abl activation at 5 months of age (Figure 4A). As we expected, diets supplemented with the c-Abl inhibitors nilotinib and neurotinib decreased p-c-Abl levels, suggesting that both treatments decreased c-Abl activation in the central nervous system (Figure 4A). To address if c-Abl inhibition is associated with an improvement in the locomotor function we evaluated the locomotor skills of mice through the Hanging test performed once a week from 4 weeks old until 11 months of age (Figure 4B). As expected, NPA mice fed with a control diet showed a significant and progressive impairment in locomotor function compared to WT mice (Figure 4B). Mice treated with neurotinib showed a modest delay in NPA-induced locomotor function impairment until 20 weeks of age. After that, the deterioration rate increased, similar to NPA mice treated with the control diet, until the end of treatment (Figure 4B). Surprisingly, we found that the NPA mice fed with the nilotinib supplemented diet maintained the locomotor function until the end of the treatment, showing very similar behaviour to WT mice.

The results were also analyzed by generating a deterioration curve with Hanging test data, which shows the percent of mice that score equal to or above three during treatment (a score less than three indicates locomotor impairment) (Figure 4C). As we expected, 100% of WT mice treated with the control diet had equal or above score 3 until the end of the treatment. Interestingly, 50% of NPA mice treated with the control diet had a score of three or higher at 10 weeks of treatment, while in the NPA mice treated with the neurotinib diet was at 21 weeks of treatment. Altogether, these results show a shift in the curve to the right for treatments with neurotinib and nilotinib, indeed the effect was bigger with the latter c-Abl inhibitor. At the end of treatment (46 weeks of treatment), 72% of NPA mice treated with the nilotinib diet and 30% of NPA mice treated with the neurotinib diet had a score 3 or higher. All of these analyses suggest that c-Abl inhibitors supplemented diets improved locomotor function and delayed deterioration, where nilotinib showed a significant and stable effect throughout the entire treatment (Figure 4C). NPA mice fed with control diet as well as NPA mice treated with diets supplemented with the c-Abl inhibitors showed similar loss of weight, starting around 5 months of age (150 days) (Supplementary Figure S6).

We next analyzed the histology of the cerebellum in mice at 5 months of age, after 4 months of treatment. We observed that the NPA cerebellum is smaller than the WT cerebellum at this age, indicating structural alterations in NPA mice. Furthermore, we found an impressive and significant loss of Purkinje neurons, followed by calbindin immunohistochemistry, at anterior and posterior lobules in the NPA mice cerebellum (Figure 4D). Interestingly, in accordance with our results using imatinib (acute treatment), we found that NPA mice treated with the nilotinib supplemented diet showed a significant increase in neuronal survival at posterior lobules 7 and 8, whereas mice treated with a neurotinib supplemented diet showed a trend for an increase in neuron survival that was not significant. Interestingly, this correlates with a better improvement in locomotor function with the nilotinib treatment compared with the neurotinib treatment (Figure 4D).

### Chronic c-Abl Inhibition Treatment Improves Cognitive Decline and Decreases Brain Neuronal Disorganization and Gliosis in NPA Mouse Brains

**FIGURE 5:**
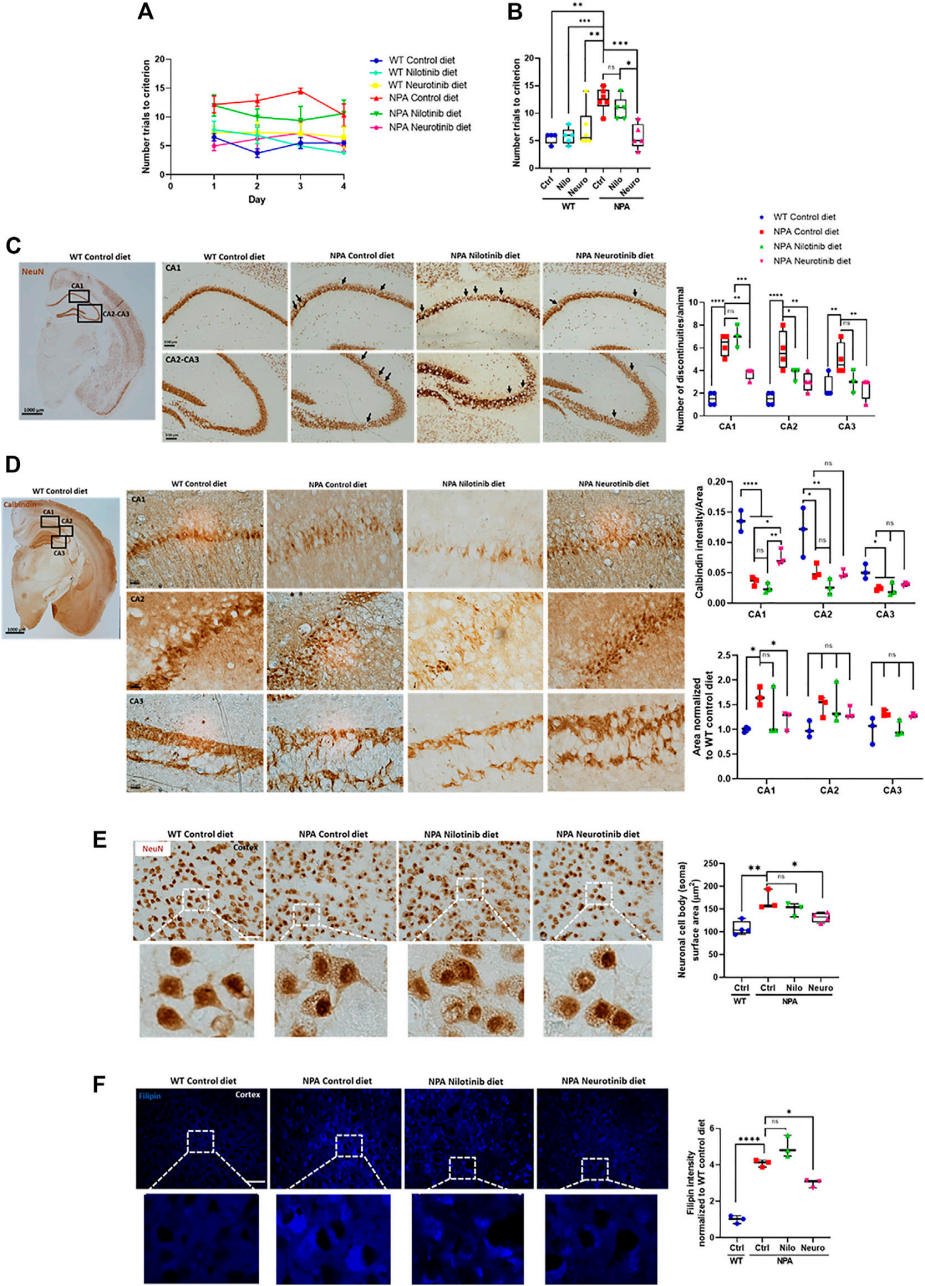
Chronic c-Abl inhibition treatment improves cognitive decline and decreases brain neuronal disorganization in NPA mice. WT and NPA mice received nilotinib (200 ppm) and neurotinib (67 ppm) supplemented diets or control diet starting at p21 until 7 months of age. **(A)** Memory flexibility test was used to evaluate cognitive functions. Graphs show the number of trials every day during the test **(A)** and the average among 4 days of test **(B)**. The number of animals was: WT control (Ctrl) diet = 4; WT nilotinib (Nilo) diet = 5; WT neurotinib (Neuro) diet = 6; NPA control diet = 6; NPA nilotinib (Nilo) diet = 5; NPA neurotinib (Neuro) diet = 5. ANOVA, Tukey *post-hoc*: *p < 0.05; **p < 0.01; ***p < 0.001 **(C)** Coronal sections of WT and NPA brain were stained with anti-NeuN antibody and 3,39-diaminobenzidine as chromogen. The rectangles in the first photo indicate where the magnification shown in the following photos comes from. Arrows point to actual discontinuities. Graph bars indicating discontinuity differences between WT and NPA mice with treatment in hippocampal subfields CA1, CA2, and CA3. Data are shown as the number of discontinuities/animal. The following number of animals was used: WT control = 4; NPA control = 4; NPA nilotinib = 3; NPA neurotinib = 4. ANOVA, Tukey *post-hoc*: *p < 0.05; **p < 0.01; ***p < 0.001; ****p < 0.0001. **(D)** Coronal sections were stained with calbindin which is a member of the large EF-hand family of calcium-binding proteins. These staining methods evidenced well-defined layers in the cortex and hippocampus structure. Neuronal disorganization with a decreased number of neurons is evident. The rectangles in the first photo indicate where the magnification shown in the following photos comes from. Image representative is shown. The following number of animals was used: WT control = 3; NPA control = 3; NPA nilotinib = 3; NPA neurotinib = 3. ANOVA, Tukey *post-hoc*: *p < 0.05; **p < 0.01; ****p < 0.0001. **(E)** Cortex neurons of the brains from WT and NPA mice were stained with NeuN antibody and 3,39-diaminobenzidine as chromogen. The area of the neuronal body was measured. 50 cells were measured by each mouse. The following number of animals was used: WT control (Ctrl) = 4; NPA control (Ctrl) = 3; NPA nilotinib = 3; NPA neurotinib = 4. ANOVA, Tukey *post-hoc*: *p < 0.05; **p < 0.01. **(F)** Slices were stained with Filipin staining to evaluate lipid accumulation. The following number of animals was used: WT control (Ctrl) = 3; NPA control (Ctrl) = 3; NPA nilotinib = 3; NPA neurotinib = 3. Image representative. ANOVA, Tukey *post-hoc*: *p < 0.05; ****p < 0.0001. Scale bar = 50 μm. In the box-and-whisker plots, the center line denotes the median value, edges are upper and lower quartiles, whiskers show minimum and maximum values and points are individual experiments.

Cerebellum damage in NPA disease has been well described, however, less is known about the pathological changes in other CNS regions. A previous report shows that cortex and hippocampus are affected in NPA pathology and associated with impairment in learning and memory (Arroyo et al., 2014). To asses the contribution of c-Abl in the cognitive impairment, we performed the Memory flexibility test, which is a modified Morris water maze test (Chen et al., 2000; Vorhees and Williams, 2006; Toledo and Inestrosa, 2010) in NPA mice at 7 months of age. The number of trials to reach the platform was used to evaluate cognitive function. As we expected, we found that NPA mice treated with control diet took significantly more trials to learn where the platform is, in comparison to WT mice during each day of testing (Figure 5A) or as an average of all days (Figure 5B), confirming a cognitive impairment in NPA mice at this age. Interestingly, NPA mice treated with a nilotinib supplemented diet showed a tendency toward improved cognitive function while the NPA mice treated with neurotinib supplemented diet showed a significant improvement in cognitive function in comparison to NPA mice treated with control diet, producing results similar to WT mice. Therefore, c-Abl inhibition improves cognitive functions of NPA mice and these results suggest that neurotinib is a better treatment than nilotinib for the cognitive alterations in NPA disease (Figure 5B).

After the memory flexibility test, mice were sacrificed and examined for neuronal damage and changes in the neuronal organization in the brain cortex and hippocampus. We stained tissues for neuronal-specific markers, the DNA-binding protein NeuN and the calcium-binding protein calbindin, which show well-defined layers in the cortex and hippocampus structure. NPA mice exhibited discontinuities in hippocampus zones CA1, CA2, and CA3 using NeuN immunostaining. Quantitation of the effect showed more discontinuities in the dorsal hippocampus of NPA mice (Figure 5C). Moreover, calbindin staining of the hippocampus from NPA mice also showed an increase of the zone area with a decrease in calbindin staining, suggesting structural disorganization and a decrease in number of calbindin positive neurons in the CA1 zone (Figure 5D). In addition, in the NPA mice cortex, the cell body of neurons was significantly bigger than the cell body of neurons from WT mice (Figure 5E). This correlated with a significant increase in Filipin staining levels in the cortex from NPA mice, confirming cholesterol accumulation in the NPA brain (Figure 5F). Interestingly, discontinuities and disorganization decreased in NPA mice treated with neurotinib and nilotinib supplemented diets, suggesting that c-Abl inhibition restores proper structuring and organization of the brain (Figures 5C,D). Additionally, c-Abl inhibition using only neurotinib diet treatment decreased neuronal cell body surface area and lipid accumulation, supporting the participation of c-Abl activation in ALP alterations (Figures 5E,F).

**FIGURE 6:**
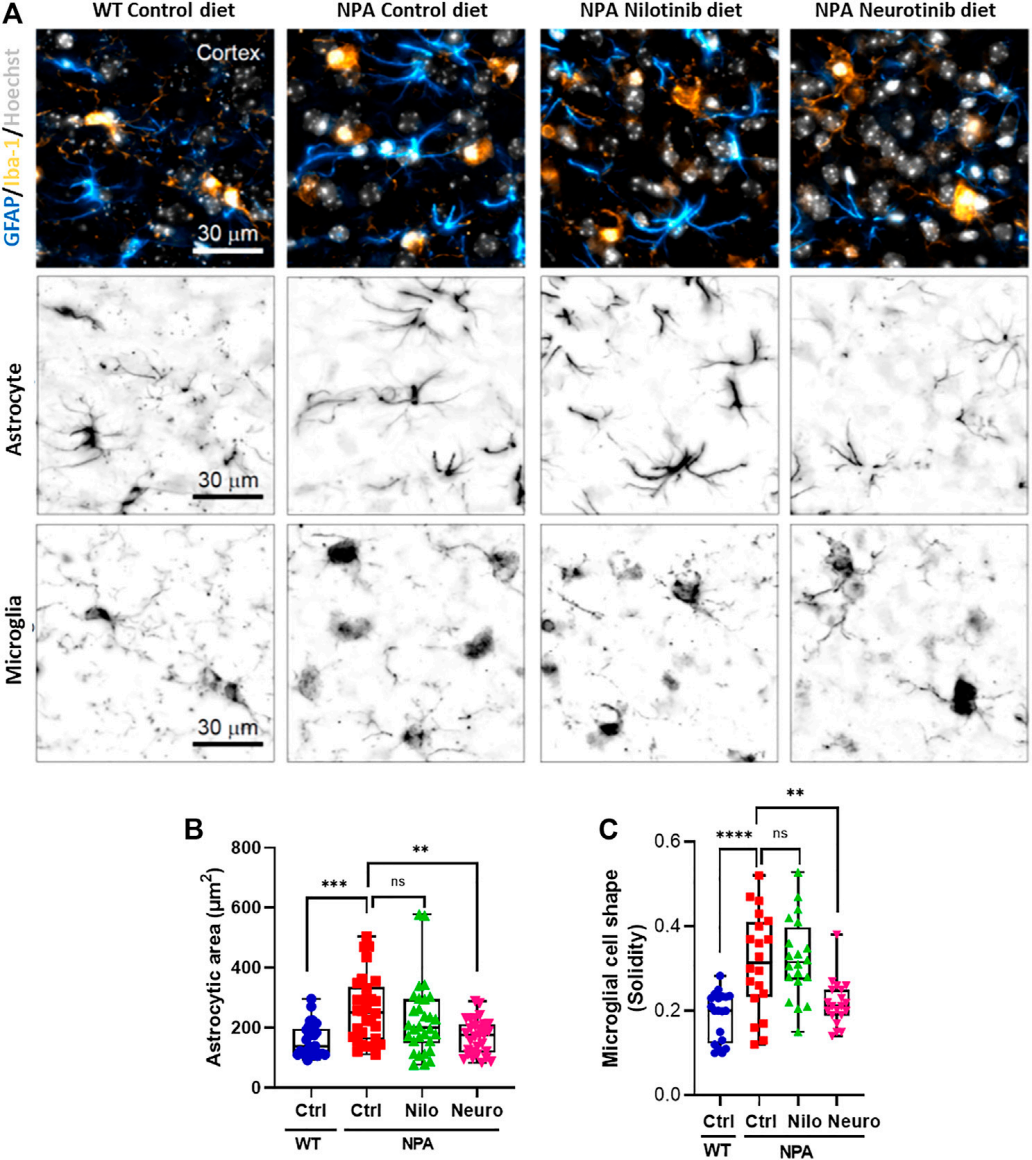
Chronic c-Abl inhibition decreases glial activation in NPA mice. WT and NPA mice received nilotinib (200 ppm; 30 mg/kg) and neurotinib (67 ppm; 10 mg/kg) supplemented diets or control diet starting at p21 until 7 months of age. **(A)** Markers of astrocyte (GFAP) and microglia (Iba-1) were analyzed by immunofluorescence in slices of brain from WT and NPA mice. Confocal images were obtained of the cortex for each condition. A representative image of the cortex is shown in the first row; astrocytes (cyan hot), microglia (orange hot), and nucleus (grays). Representative images to visualize astrocyte and microglial shape are shown in the second and third row, respectively. **(B)** Astrocyte area was measured from GFAP positive cells. For each condition, *n* = 10 cells were measured by animal; three mice/group. ANOVA, Tukey *post-hoc*: **p < 0.01, ***p < 0.001. **(C)** Microglia shape was determined from Iba-1 fluorescence. For each condition, *n* = 5–10 cells were measured by animal; three mice/group. ANOVA, Tukey *post-hoc*: **p < 0.01, ****p < 0.0001. In the box-and-whisker plots, the center line denotes the median value, edges are upper and lower quartiles and whiskers show minimum and maximum values.

Previous studies have shown microglial activation in NPA disease (Gabande-Rodriguez et al., 2019). As expected, we found astrocyte and microglial activation in the cortex of NPA mice (Figure 6A). Interestingly, we found that the treatment with neurotinib reduced astrocyte activation, decreasing the size of astrocytes in the brains of NPA mice treated with neurotinib (Figure 6B; Supplementary Figure S1F). Moreover, we analyzed the cell shape of microglia as an index of activation, as resting-state microglia are ramified whereas activated microglia have an amoeboid form. Treatment with neurotinib restored the microglial shape in NPA brains, and the morphology of those cells is similar to that observed in WT brains (Figure 6C; Supplementary Figure S1G). These data show that c-Abl inhibition using neurotinib reduces both astrogliosis and microgliosis.

Altogether these results show that c-Abl inhibition decreases Purkinje cell death in the cerebellum correlating with an improvement in autophagy flux and locomotor function. Furthermore, our results show that there is glial activation and neuronal disorganization in NPA mice brains, which correlate with impairment of the cognitive function. Treatment with c-Abl inhibitors supplemented diets reduced these alterations improving memory and locomotor function in NPA mice. Our results strongly support the potential use of c-Abl inhibitors for clinical treatment of NPA patients.

## Discussion

Our work represents the first demonstration that c-Abl kinase plays an important role in neurodegeneration that is a hallmark of NPA pathology and that c-Abl inhibition can improve neurological decline of NPA mice. The major findings of this work are the following: 1) c-Abl kinase is activated in several NPA models; 2) *In vitro* NPA models show autophagy and lysosomal alterations; 3) c-Abl inhibition induces autophagy flux and lowers sphingomyelin accumulation in *in vitro* NPA models and 4) c-Abl inhibition associates with a decrease in neuronal death, brain neuronal disorganization, glial markers and with an improvement in locomotor and cognitive functions in NPA mice.

We found an increase of p-c-Abl protein levels and an increase in its nuclear localization in human NPA fibroblasts and NPA mouse primary neurons. Interestingly, c-Abl nuclear localization has been related to its pro-apoptotic functions, leading to cellular death (Wen et al., 1996). c-Abl is a non-receptor tyrosine kinase that has different biological functions depending on the cell type and can regulate several pathways in response to different signals (Wang, 2014). It has one nuclear export signal (NES) and three nuclear localization signal (NLS) motifs in its C-terminus consistent with its cytoplasmic and nuclear localization and its capacity to regulate gene expression through c-Abl substrates such as the transcription factors p73 (Klein et al., 2011), TFEB (Contreras et al., 2020) and histone deacetylase HDAC2 (Gonzalez-Zuniga et al., 2014). Therefore, it is possible that in the NPA pathology the c-Abl kinase activation affects the expression of different genes. This possibility deserves further investigation.

c-Abl has been reported to be activated in other lysosomal and/or neurodegenerative diseases, including NPC disease, Gaucher disease (Yanez et al., 2021), Alzheimer’s, and Parkinson’s disease (Alvarez et al., 2004; Alvarez et al., 2008; Imam et al., 2011; La Barbera et al., 2021). Interestingly, NPC disease is a lysosomal storage and neurodegenerative disorder that shares several characteristics with NPA disease despite they are caused by mutations in different genes (Yanez et al., 2020). In NPC disease, the c-Abl signaling pathway impacts several of its downstream targets, including the p73 transcription factor (Alvarez et al., 2008), HDAC2 (Gonzalez-Zuniga et al., 2014; Contreras et al., 2016), APP (Yanez et al., 2016) and TFEB (Contreras et al., 2020). It would be interesting to study if these signaling pathways are also activated and participate in NPA pathological mechanisms. Thus, these diseases, which are different in their etiology, could share a common mechanism for neuronal death that involves activation of the c-Abl kinase.

It has been described that c-Abl kinase can be activated in response to distinct types of cellular stress (Sun et al., 2000; Shaul and Ben-Yehoyada, 2005; Hopkins et al., 2012). However, the upstream stimulus and mechanism that activates c-Abl kinase in NPA disease remain unclear. The c-Abl kinase signaling activation in NPC neurons has been linked to increments in ROS levels (Klein et al., 2011). Interestingly, increased oxidative stress has also been described in NPA disease (Perez-Canamas et al., 2017). On the other hand, some studies suggest that c-Abl may be regulated in different cellular contexts by lipids (Van Etten, 2003). Therefore, there is a possibility that sphingomyelin or other lipids accumulation could activate c-Abl kinase in NPA pathology. Clearly, we have more to learn about the regulation of this kinase. This is an interesting topic that remains to be elucidated.

As previously mentioned, we utilized several NPA models including a pharmacological NPA model using desipramine which induces functional inhibition of ASM. The ASM enzyme is attached by electrostatic forces to the inner lysosomal membrane, thereby being protected against proteolysis. High concentrations of the protonated bases, such as desipramine, disturb the binding of ASM to the inner lysosomal membrane and result in detachment of ASM and subsequent inactivation possibly involving proteolysis (Kornhuber et al., 2010). However, considering desipramine is a promiscuous tricyclic antidepressant (TCA), we cannot rule out that part of the observed effect of c-Abl activation is produced through other targets of desipramine. Nevertheless, it is interesting that the ASM inhibition leads to c-Abl activation indicating that lipids homeostasis alterations are related to c-Abl regulation.

Autophagy is an important cellular process that eliminates damaged proteins, dysfunctional organelles, and protein aggregates, where lysosomes have a central role (Menzies et al., 2017). Neurons are particularly affected by disruptions of autophagy which are associated with many neurodegenerative disorders (Nixon, 2013; Lee et al., 2016; Menzies et al., 2017). It has been published that autophagy can be regulated by c-Abl kinase (Hebron et al., 2013; Contreras et al., 2020; Karim et al., 2020; La Barbera et al., 2021). Interestingly, we found that activated c-Abl levels and autophagy markers are increased in NPA cellular models. Furthermore, we found a high number of autophagy p62 positive vesicles around the nucleus. Our results are in accordance with what has been published before, fibroblasts from NPA patients accumulate elongated and unclosed autophagic membranes, as well as abnormally swollen autophagosomes (Corcelle-Termeau et al., 2016). Moreover, it has been described that autophagosome clearance is delayed leading to the accumulation of vesicles in other similar pathologies. For example, alterations in lysosomal function and autophagy are tightly associated with neurodegeneration in NPC disease (Liao et al., 2007) and other neurodegenerative disorders such as Parkinson's disease (Garcia-Sanz et al., 2018), Gaucher disease (Aflaki et al., 2016), and Alzheimer's disease (Cermak et al., 2016), among others (Lee et al., 2016; Menzies et al., 2017). In this sense, it has been described that lipid accumulation in NPA could contribute to autophagosomes accumulation because of autophagosome-lysosome fusion impairment (Li et al., 2014; Corcelle-Termeau et al., 2016). Moreover, lysosomal membrane permeabilization leading to the cytosolic release of lysosomal enzymes, such as Cathepsin B, has been described in NPA fibroblasts (Gabande-Rodriguez et al., 2014) and also in NPC models by our group (unpublished results).

We found that NPA cells exhibit alterations in lysosomal function and autophagy as well as c-Abl activation. Our results suggest that both mechanisms are connected. We observed that when c-Abl is inhibited, there is a decrease in sphingomyelin accumulation, autophagy markers, and cellular death. Also, we found that c-Abl inhibition increases autophagosome-lysosome fusion suggesting the induction of the autophagy flux. However, we can not rule out that c-Abl inhibition could be affecting different processes and characteristics related to lysosomes, such as membrane permeability and function, among others. The mechanism and how and what stage of autophagy flux could be regulated by c-Abl is not fully understood. One option is that c-Abl could be regulating gene expression through the transcription factor TFEB (Contreras et al., 2020). Thus, it could be regulating autophagy, lysosomal biogenesis, clearance, and exocytosis (Can et al., 2011; Ren et al., 2018). Accordingly, we propose that c-Abl inhibition is regulating different processes related to lysosomes at the same time. This could explain the decrease in sphingomyelin accumulation after 24 h of treatment with c-Abl inhibitors observed in this work. This effect is similar to the effect observed in NPC cells where c-Abl inhibition reduced cholesterol accumulation (Contreras et al., 2020). Another option is that c-Abl could phosphorylate some proteins related to the actin cytoskeleton (Mitsushima et al., 2006) and autophagy proteins such as beclin (Yu et al., 2020). This could directly affect the formation of autophagy vesicles and/or their movement. As mentioned, neurons are particularly affected by disruptions of autophagy (Nixon, 2013). Therefore, an improvement in autophagy could decrease neuronal death. This could positively affect Purkinje neurons in the cerebellum, hippocampal and cortical neurons in the brain, leading to a decrease in neuronal death and an improvement in locomotor and cognitive functions, respectively, in the NPA mouse. Actually, we found that mice treated with injections of imatinib show an increase in Purkinje neuron survival and a decrease in CD68 signal associated with cerebellar inflammation when c-Abl is inhibited. These results are similar to those published in NPC mice with an imatinib treatment (Alvarez et al., 2008). Here we also demonstrate a decrease in glial activation in NPA mice treated with c-Abl inhibitors. However, more studies are necessary to evaluate the effect of c-Abl inhibition on autophagy flux and its connection with neuronal death.

NPA mice live approximately 11 months, allowing us an opportunity to study the effects of a prolonged, chronic treatment using c-Abl inhibitors supplemented diets. This strategy is less invasive and is closer to an oral treatment such as that an NPA patient might receive. Also, a longer treatment allowed us the opportunity to explore different brain areas that could be involved in the impairment of other functions, such as learning and memory in the NPA pathology. It has been well described that the cortex and hippocampus in the brain are involved in learning and memory (Miller, 2000; Preston and Eichenbaum, 2013; Opitz, 2014). Indeed, both areas before mentioned are affected in NPA pathology consistent with the impairment of the cognitive functions (Arroyo et al., 2014). Chronic treatment was performed using nilotinib and neurotinib supplemented diets. Imatinib was not considered for chronic treatment because it has a low Blood-Brain Barrier permeability (Wolff et al., 2003). Interestingly, we obtained differential results with the supplemented diets. On the one hand, our results show an outstanding improvement in the locomotor function in NPA mice treated with the nilotinib supplemented diet which correlates with an increase in Purkinje neuron survival in the cerebellum. And on the other side, we found a significant improvement in cognitive functions in NPA mice treated with neurotinib that correlated with a decrease in brain neuronal disorganization and gliosis in NPA mouse brain. Considering these results, it would be interesting to evaluate if a combined therapy has a synergistic effect.

The reasons that account for these differences between the treatments with these two c-Abl inhibitors neurotinib and nilotinib are not clear yet. Nilotinib is a classical c-Abl inhibitor that has been used in other neurodegenerative pathologies such as Parkinson’s disease (Pagan et al., 2019) and Alzheimer’s disease (Turner et al., 2020). Neurotinib is a new c-Abl inhibitor that was designed by our laboratory and the NCATS-NIH group. Nilotinib and neurotinib present different c-Abl inhibition mechanisms. Nilotinib binds to the ATP binding cleft between the N-terminal and C-terminal lobes, while neurotinib binds to an allosteric pocket for myristate at the C-terminal lobe of the kinase domain. In contrast to nilotinib which targets multiple kinases, the allosteric inhibitor neurotinib is highly selective for the c-Abl kinase. In addition, it is important to mention that the diets supplemented with the inhibitors were used with different concentrations: 67 ppm (10 mg/kg) for neurotinib, while 200 ppm (30 mg/kg) was used for nilotinib. Also, it is relevant that neurotinib has a better brain penetration and remains more time than nilotinib in the brain (Supplementary Figure S5B). Thus the concentrations of neurotinib and nilotinib for efficacy experiments were chosen to provide proper brain levels of the drug, where 200 ppm allows a reasonable concentration of nilotinib in the brain. A possible explanation for the differences in the observed results between these two compounds is that 200 ppm of nilotinib would provide better exposure toward the peripheral nervous system and muscle, which has been described showing functional defects in NPA mice (Michailowsky et al., 2019), while neurotinib distribute with better efficacy toward the CNS. More experiments are required to better understand pharmacokinetic to pharmacodynamic aspects of c-Abl inhibition. Although we obtained promising results, we did not observe an increase in the survival of NPA mice using a small number of animals, unlike a recent paper published where the authors found an increase in survival modulating the endocannabinoid signaling (Bartoll et al., 2020). It is possible that we need to increase the number of animals to obtain better results but also it could be that an increase in survival requires an integral effect including brain and peripheral organs.

Recent reports show that c-Abl inhibitors are being used in clinical trials for different neurodegenerative pathologies, including Parkinson’s disease (Abushouk et al., 2018; Pagan et al., 2019) and other Dementias (Pagan et al., 2016), Huntington’s disease (Clinical trial gov identifier NCT03764215) and Alzheimer’s disease (NCT02947893) (Turner et al., 2020). Results in Parkinson’s patients are promising because they show improvement in locomotor function and decreased synuclein accumulation, stimulating a new phase of this study (Pagan et al., 2019).

Considering these antecedents and our results, c-Abl is a promising therapeutic target for NPA. Moreover, c-Abl inhibitors are safe drugs that are well tolerated, with mild secondary effects already approved by the FDA for the treatment of chronic myeloid leukemia and another kind of cancers. Altogether, our work opens new perspectives for therapeutic interventions supporting the potential use of c-Abl inhibitors for the clinical treatment of NPA patients.

## Data Availability

The original contributions presented in the study are included in the article/Supplementary Material, further inquiries can be directed to the corresponding authors.
