# Supplementary figures and images for: c-Abl Activation Linked to Autophagy-Lysosomal Dysfunction Contributes to Neurological Impairment in Niemann-Pick Type A Disease
Source: Front Cell Dev Biol. 2022 Mar 18;10:844297. doi: 10.3389/fcell.2022.844297 (PMC8985125; doi:10.3389/fcell.2022.844297)

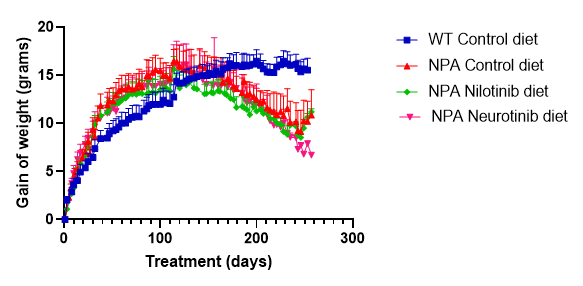

Supplement: Supplementary file 1 [file Image6.TIF]

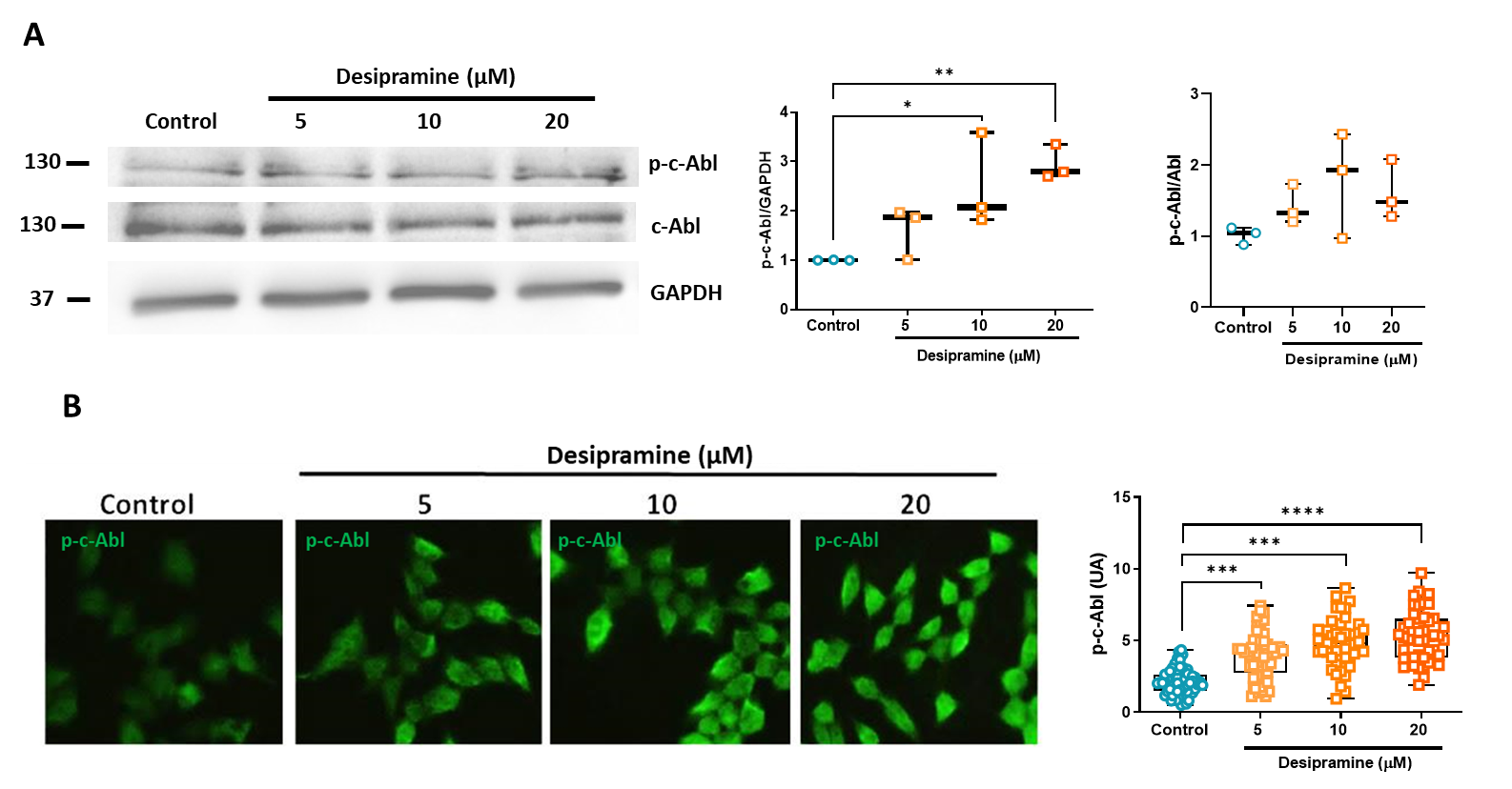

Supplement: Supplementary file 2 [file Image3.TIF]

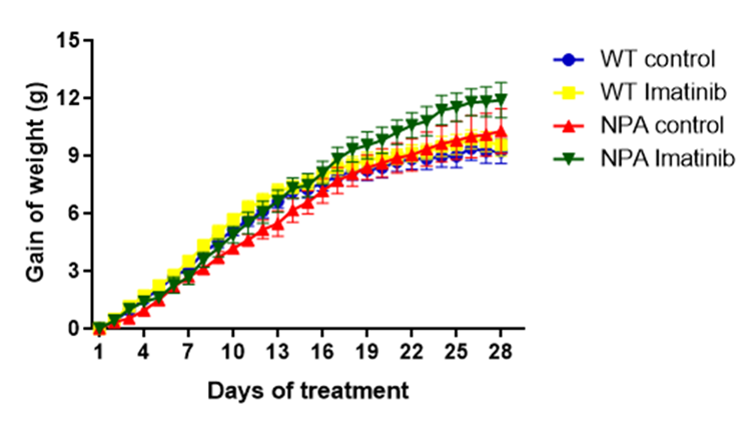

Supplement: Supplementary file 3 [file Image4.TIF]

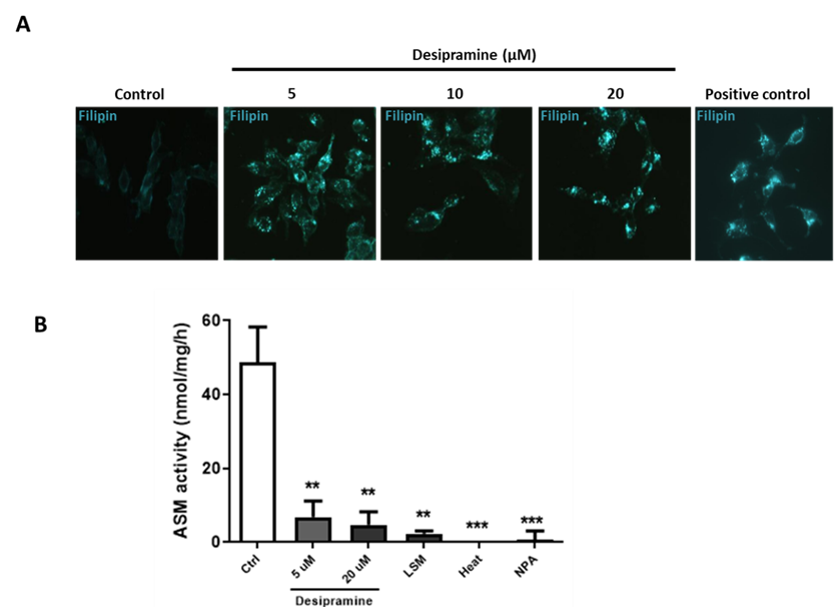

Supplement: Supplementary file 4 [file Image2.TIF]

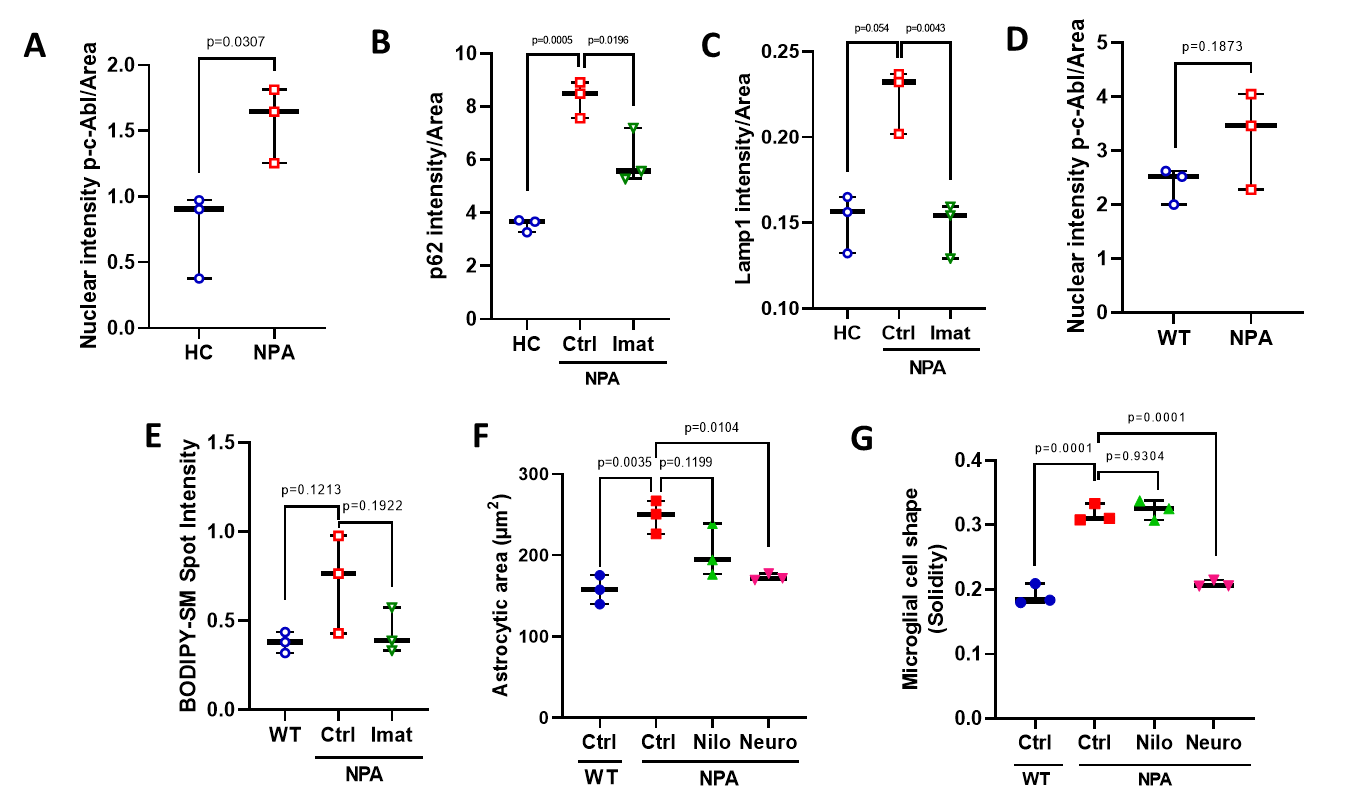

Supplement: Supplementary file 5 [file Image1.TIF]

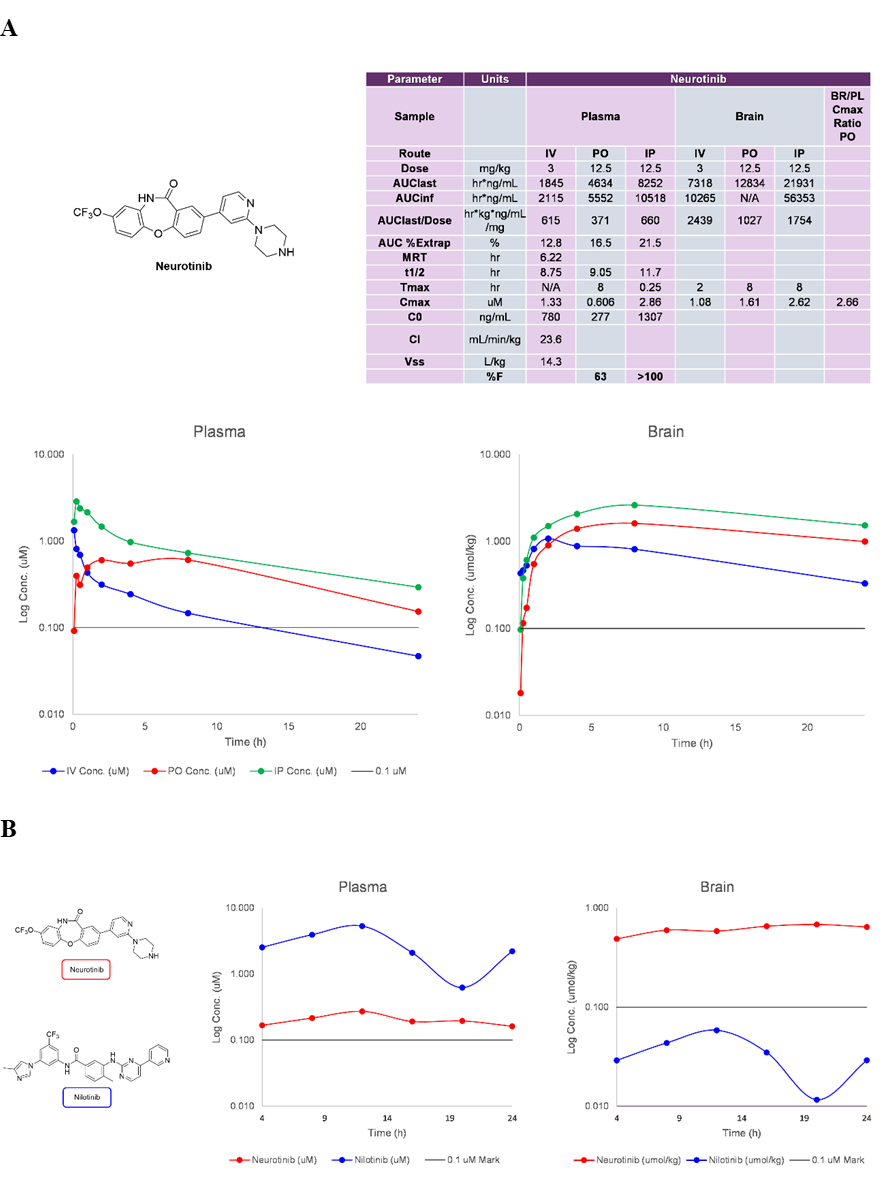

Supplement: Supplementary file 7 [file Image5.TIF]
