# Supplementary material for: c-Abl Activation Linked to Autophagy-Lysosomal Dysfunction Contributes to Neurological Impairment in Niemann-Pick Type A Disease
Source: Front Cell Dev Biol. 2022 Mar 18;10:844297. doi: 10.3389/fcell.2022.844297 (PMC8985125; doi:10.3389/fcell.2022.844297)
Supplement: Supplementary file 6 [file DataSheet1.pdf]

Supplementary Figure 1. Graphs of the immunofluorescence quantifications by experiment. (A) HC and NPA fibroblasts were fixed and immunostained using an anti-p-c-Abl Tyr412 antibody (green) and Hoechst staining for nucleus (blue). For each condition, three independent experiments were performed; 15 cells /experiment were analyzed. Student's t-test. (B) HC and NPA fibroblasts were treated with imatinib (10 µM) for 24 h, fixed, and immunostained using an anti-p62 antibody (green) and (C) anti-Lamp1 (green). Hoechst staining for the nucleus (blue). For each condition, three independent experiments were performed; 10-18 cells by experiment were analyzed. ANOVA, Tukey post-hoc. (D)) Primary neurons were 7 days in vitro, fixed, and immunostained using anti-p-c-Abl Tyr412 antibody (red) and Hoechst staining for nucleus (blue). For each condition, three independent cultures were performed; 10-20 neurons were measured by culture. Student's t-test. (E) Primary hippocampal neurons were treated with imatinib 5 µM by 24 h. Sphingomyelin accumulation was analyzed by BODIPY-SM. For each condition, three independent cultures were performed; 10-20 neurons were measured by culture. (F) WT and NPA mice received nilotinib (200 ppm) and neurotinib (67 ppm) supplemented diets or control diet starting at p21 until 7 months of age. Astrocyte area was measured from GFAP positive cells. 3 mice were used per group and 10 cells were analyzed per mouse. (G) Microglia shape was determined from Iba-1 fluorescence. 3 mice were used per group and 5-10 cells were analyzed per mouse. ANOVA, Tukey post-hoc. In the box-and-whisker plots, the center line denotes the median value, edges are upper and lower quartiles and whiskers show minimum and maximum values and points are individual experiments or number of animals used.

Supplementary Figure 2. Characterization of an NPA neuronal pharmacologic model. SHSY5Y cells were treated with 5, 10 and 20 µM of desipramine for 24 h. (A) Cholesterol accumulation was detected with Filipin staining. As a positive control we used cells treated with U18666 (0.5 µg/mL), which induces cholesterol accumulation. (B) ASM activity was measured through a fluorimetric method in SHSY5Y cells treated with desipramine 5 µM and 20 µM. Other controls include: LSM, a competitive substrate, heated proteins, NPA human fibroblasts and vehicule (Ctrl). Mean ± SEM of three independent experiments are shown. $^{**}p \leq 0.01$; $^{***}p \leq 0.001$.

Supplementary Figure 3. c-Abl is activated in a pharmacological NPA model. SHSY5Y cells were treated with 5, 10 and 20 µM of desipramine for 24 h. p-c-Abl levels were analyzed by western blot (A) and immunofluorescence (B). (A) Three independent experiments were performed. (B) For each condition, n=15-20 neurons were measured by experiment; three independent experiments. ANOVA, Tukey *post-hoc*: $^{*}p \leq 0.05$; $^{**}p \leq 0.01$; $^{***}p \leq 0.001$;$^{****}p < 0.0001$. In the box-and-whisker plots, the center line denotes the median value, edges are upper and lower quartiles and whiskers show minimum and maximum values.

Supplementary Figure 4. Weight gain in mice treated with i.p injections of imatinib. WT and NPA mice were treated with imatinib and vehicle from 3 weeks of age until 7 weeks of age. Gain of weight was recorded during all treatment. The numbers of animals was: WT control=11; WT imatinib=10; NPA control =8; NPA imatinib=9.

Supplementary Figure 5. Pharmacokinetics and distribution of c-Abl inhibitors. (A) Pharmacokinetic parameters of single dose intra venous (IV), oral administration (PO) and intra peritoneal (IP) administration of neurotinib in plasma and brain. (B) Distribution after 14-day administration of chow diet containing 67 ppm (10mg/kg) neurotinib and 200 ppm (30 mg/kg) nilotinib in plasma and brain (B).

Supplementary Figure 6. Weight gain during chronic treatment with c-Abl inhibitor supplemented diets. WT and NPA mice were treated with nilotinib and neurotinib supplemented diets from 3 weeks of age until 11 months of age. Gain of weight was recorded during all treatment. The numbers of animals was: WT control diet=10; NPA control diet=:10; NPA Nilotinib diet=11; NPA Neurotinib diet=10.
